# The context dependency of multiple stressor effects on biodiversity: A commentary on Kefford et al. (2022)

**DOI:** 10.1111/gcb.16483

**Published:** 2022-11-03

**Authors:** Andrew P. Beckerman

**Affiliations:** ^1^ School of Biosciences, Ecology and Evolutionary Biology The University of Sheffield Sheffield UK

Biodiversity on the planet underpins numerous functions and services central to humanity including carbon capture, food production and freshwater (Millenium Ecosystem Assessment, 2005). Biodiversity is also just beautiful and the myriad of species, colours and forms that surround us make life more pleasurable. But there is no doubt that biodiversity is at risk around the planet. The diversity, mode, magnitude and frequency of perturbations to ecosystems we rely on is increasing. Climate change, environmental change and land‐use change continue to put pressure on biodiversity that we rely on, to the point that the planet may be entering a 6th Mass Extinction (Ceballos et al., 2015; IPBES, 2019; IPCC, 2022).

As a result, being able to predict the timing, locations and magnitude of impacts on biodiversity of multiple stressors ranks as one of the most important research agendas. This agenda is not simply a set of interesting ecological questions, for the answers are relevant to nearly all sustainable development goals (UN, 2020). Being able to make such predictions about biodiversity loss is central to driving policy to stem future losses of biodiversity and mitigate those that are already going to happen.

Over the past several decades, great strides have been made in defining the various ways in which multiple, simultaneously acting threats to biodiversity might manifest (Folt et al., 1999; Halpern & Fujita, 2013; Orr et al., 2020; Piggott et al., 2015; Schäfer & Piggott, 2018; Simmons et al., 2021). At the heart of this are statistical toolboxes that can differentiate between two large classes of effect: additive effects and non‐additive effects (also known as interactions or context‐dependent effects). When stressor effects combine simply by adding them up—for example, if stressor A causes a 10 unit decline and stressor B a 20 unit decline and their combined effect is a 30 unit decline—the combined effect is additive. In contrast, non‐additive (interaction) effects arise when the combined effect of stressors is more—synergistic—or less—antagonistic—than would be expected by simply summing the effects.

Additive effects are easier to manage and mitigate because such a relationship implies that one need only know about how each individual stressor drives a change. However, when there is an interaction, it means that the effect of one stressor on, for example biodiversity, depends on the level of the other stressor. There is non‐additivity, a potential for synergy or antagonism, and clear context dependence. Developing policy for and managing context dependency is much more challenging because just knowing the independent effects of each stressor is no longer sufficient (Simmons et al., 2021).

Substantial advances have been made in statistical tools and mathematical modelling to make inference and draw conclusions about far more nuanced outcomes in the face of multiple stressors (see Simmons et al., 2021 for overview). However, several major challenges remain to making effective predictions and guiding policy. First is the burden of two stressors. Most empirical research and statistical examples still focus on no more than two stressors. Second is the burden of biological diversity. While we truly need to assess the impacts of multiple simultaneous threats on biodiversity, theory and empirical work remains constrained, for many valid reasons (it's hard!) on one or a few species and their responses. The ecological scale for biodiversity—the community—is not the default scale for current research. We are simply not studying the impacts of multiple stressors on biodiversity as effectively as we need to. Third is the burden of space and time. Context dependence—when the effect of one stressor varies by the value/level of another—implies either spatial or temporal variation in exposure to and impact of stressors. It is relatively ‘easy’ to create gradients in the laboratory or in a model. It is much less easy to evaluate the spatial and temporal foundation of this context dependence, despite this being so very necessary to predict the timing, locations and magnitude of impacts on biodiversity of multiple stressors. Finally, there is the burden of ecological and evolutionary time. Typical experiments on multiple stressors rarely extend beyond a single season and a single ‘exposure’ event when, in reality, biodiverse communities are persisting or declining amidst multiple stressors over long periods of time.

The research article by Kefford et al. in this issue joins a small but growing number of studies (e.g. Rillig et al., 2019) that aim to address these burdens. Kefford et al. address all four burdens by taking advantage of a large dataset that comprises detail on family richness in three orders of insects that use freshwaters. The data are spatially resolved across a substantive latitudinal gradient spanning 3600 km. There are two major habitat types (edges and riffles) and samples span 2000 m in elevation. Finally, the data on biodiversity across this spatial extent are linked to four stressors that can impact on biodiversity that also vary spatially. The stressors are salinity (defined by conductivity), turbidity, temperature and terrain slope. Salinity and turbidity in their Australian system are linked strongly to land‐use modification and particularly agriculture. Temperature varies across the latitudinal and elevation gradients in their study system. Finally, the terrain slope is a proxy for flow characteristics and velocity clearly linked to contrasts between steep rocky streams and floodplains, but also a surrogate for extreme meteorological events that are predicted to increase in frequency (IPCC, 2022) and likely lead to floodplains experiencing conditions more akin to steep mountain forces.

Kefford et al. harness these data into a clever application of generalized additive statistical models that ultimately lend themselves to asking a simple question: Does the (potential) interaction (e.g. synergy/antagonism) between salinity and turbidity vary by temperature and does this (potential) variation vary by flow regime and habitat. Yes, this is a potential five‐way interaction, and it is complicated. But it is the necessary type of investigation that can lead to understanding whether we can predict the timing, locations and magnitude of impacts on biodiversity of stressors.

What Kefford et al. found is, in some respects, not surprising: the interaction between salinity and turbidity does exist, but it takes both synergistic and antagonistic forms depending on habitat, temperature and other landscape variables. Embedded in their data is a strong effect of salinity on biodiversity that varies substantially with temperature, highlighting how predicting and managing the effects of land use change on biodiversity may be increasingly challenging as temperatures change.

One might argue that their overarching take home message—that there is no consistency among even the relatively simple two‐way interactions—is cause for despair. Current theory and experimental approaches that have led to core definitions of additive and non‐additive effects appear insufficient in the face of more than two stressors defined on a landscape and linked to a response variable as complex and important as biodiversity. Unless we have measured everything together, the data here might suggest we will be unable to make predictions without massive investment around the world and unable to move towards generalizing our understanding of how stressors combine. This is a perfect storm for policy and management where the uncertainties and context dependency from robust data analysis might still lead to inaction and failure to address sustainable development goals.

Keffert et al., however, make several recommendations that can move us forward from this predicament, specifically focusing on how to evaluate and estimate the scale at which consistency of relationships might emerge. They suggest focusing on the presence and distribution of higher‐order interactions, rather than form of the interactions. They also suggest focusing on and synthesizing geographically distributed sets of experiments focused on biodiversity to complement observational data analyses like theirs. And they suggest focusing on variation in the magnitude of impacts and the distribution of interaction types to guide decision‐making. Such a strategy posits, for example, that if all interactions are synergistic, it means it is necessary to aim to reduce all stressors, whereas a more nuanced approach is necessary if there is a mix of antagonism, synergy and additivity.

Ultimately, just because we see variation, context dependency and higher‐order interactions, as we do in Keffert et al.'s spatially resolved data, it does not mean there is not an ecological, spatial or temporal scale where impacts and policy decision‐making can be generalized and made effective. This is what we must aim for.

## FUNDING INFORMATION

APB and laboratory are funded by NERC‐UKRI Grants NE/S001395/1 and NE/T003502/1.

## Data Availability

Data sharing is not applicable to this article as no new data were created or analyzed in this study.
